# The Most Effective Amount of Forward Movement for Oral Appliances for Obstructive Sleep Apnea: A Systematic Review

**DOI:** 10.3390/ijerph16183248

**Published:** 2019-09-04

**Authors:** Yuki Sakamoto, Akifumi Furuhashi, Eri Komori, Hiroyuki Ishiyama, Daichi Hasebe, Kazumichi Sato, Hidemichi Yuasa

**Affiliations:** 1 Department of Oral Surgery, Hironokogen Hospital, 3-1-1 Kitayamadai Nishi-ku Kobe-shi, Hyogo 6512215, Japan; 2 Department of Oral and Maxillofacial Surgery, Aichi Medical University, 1-1 Yazakokarimata Nagakute-shi, Aichi 4801103, Japan; 3 Division of Medicine for Function and Morphology of Sensor Organ, Dentistry and Oral Surgery, Osaka Medical College. 2-7 Daigaku-machi Takatsuki-shi, Osaka 5698686, Japan; 4 Orofacial Pain Management, Graduate School of Medical and Dental Sciences, Tokyo Medical and Dental University (TMDU), 1-5-45 Yushima Bunkyo-ku, Tokyo 1138510, Japan; 5 Division of Reconstructive Surgery for Oral and Maxillofacial Region, Department of Tissue Regeneration and Reconstruction, Niigata University Graduate School of Medical and Dental Sciences, 2-5274 Gakkocho-Dori, Cyuo-ku, Nigata-shi, Nigata 9518514, Japan; 6 Department of Oral Medicine, Oral and Maxillofacial Surgery, Tokyo Dental College, 5-11-13 Sugano Ichikawa-shi, Chiba 2728513, Japan; 7 Department of Oral and Maxillofacial Surgery, National Hospital Organization Toyohashi Medical Center, 50 Imure-chou Aza Hamamichi-Ue, Toyohashi-shi, Aichi 4408510, Japan

**Keywords:** oral appliance, mandibular protrusion, obstructive sleep apnea, systematic review

## Abstract

This systematic review clarifies the amount of effective protrusion in mandibular advancement devices of oral appliances required for obstructive sleep apnea (OSA). The systematic review adhered to the Preferred Reporting Items for Systematic Reviews and Meta-Analysis (PRISMA) guidelines. Review Manager 5 and GRADEpro were used to combine trials and analyze data. The present review included three studies. In mild to moderate OSA cases, measured using the apnea–hypopnea index (AHI), 50% protrusion was more effective than 75% protrusion. However, 75% protrusion was more effective for severe cases. Sleep stage, Epworth Sleepiness Scale (ESS), snoring index, and side effects significantly differed between the groups. Additionally, 75% protrusion was more effective (AHI: 0.38, 95% CI: −0.89 to 1.65, *p* = 0.56; sleep stage 3: −1.20, 95% CI: 9.54–7.14, *p* = 0.78; ESS: 1.07, 95% CI: −0.09 to 2.24, *p* = 0.07; snoring index: 0.09, 95% CI: 0.05–0.13, *p* < 0.05; side effects: RR: 1.89, 95% CI: 0.36–9.92, *p* = 0.45). As per the AHI, 75% protrusion was effective in severe cases, whereas 50% protrusion was effective in moderate cases. Analysis of different surrogate outcomes indicated that 75% protrusion was more effective. Further, well-designed, larger trials should determine the benefits for patients. Additionally, investigations of adherence and side effects with long-term follow-up are needed.

## 1. Introduction

More than 3 million people have sleep disorders such as obstructive sleep apnea (OSA), which also includes latent sleep apnea. OSA has been shown to be associated with hypertension, depression, and lifestyle-related diseases [1,2,3]. OSA is commonly treated with continuous positive airway pressure (CPAP) or oral appliances (OAs). CPAP machines are usually prescribed to patients with an apnea–hypopnea index (AHI) between moderate and severe. OAs created by dentists are often prescribed for mild to moderate cases. In Japan, OA therapy is provided through the National Health Insurance and appliances such as monobloc and mandibular advancement devices (OAm) are often used to balance the costs. Many clinicians have set the mandibular protrusion as 50%–70% of the maximum. Initial observations in clinical settings indicate that OAs become inefficient owing to the advancement of the disease. Greater protrusion results in a larger airway that may be considered good; however, symptoms such as temporomandibular joint pain, a side effect of OAs, might appear and complicate the situation. Bartolucci et al. [4] reported no difference in the effectiveness of OAs with an incidence of forward protrusion of more than 50%. Nonetheless, their conclusion was drawn based on the results of an examination of the success rate of protrusion amounts in randomized control trials. As for the monobloc-type devices, adjustments are required depending on the comfort of the patient and effects on their OSA, but this adjustment is complicated and troublesome. Such adjustment is necessary to indicate the initial bite setting for clinicians. This systematic review examined the most effective protrusion of mandibular orientation of OAs afresh.

## 2. Materials and Methods 

This systematic review was performed following the Preferred Reporting Items for Systematic Reviews and Meta-Analyses (PRISMA) guidelines. The protocol for this review was registered with the international prospective register of systematic reviews (PROSPERO) with the registration number CRD42019136242.

### 2.1. Inclusion and Exclusion Criteria

#### 2.1.1. Inclusion Criteria

1. The definition of OA
  - Equipment that acquires upper and lower jaw impressions for every patient, which is thus precisely produced.
  - A device that exerts an effect by maintaining the lower jaw in the forward direction.
2. The diagnostic and therapeutic effects of OSA are determined by either polysomnography (PSG) or out-of-center sleep testing (OCST).

#### 2.1.2. Exclusion Criteria

1. Subject is under 18 years of age.
2. The device is ready-made.
3. The device exerts an effect by maintaining the tongue in the forward orientation.

#### 2.1.3. Conditions Not Included in the Definition

1. Equipment size, design
2. Material characteristics (e.g., hard, soft, and hybrid)
3. Thickness
4. Detailed design of the device (e.g., type of connector in separate type and presence of integral air hole)
5. Excluding comparison with forward movement of 0 mm (placebo)

### 2.2. Literature Search

The primary database used was MEDLINE (via PubMed), the Cochrane Central Register of Controlled Trials (CENTRAL), and Japan Medical Abstracts Society Research (Ichushi-Web). No limits were set with respect to the year of study or language. We selected only randomized controlled trials (RCTs) in the OAm design. A thorough literature search was conducted that was completed on 27 April 2019. A search strategy was executed using the keywords shown in Box 1.

Box 1 **Keywords used for the literature search.**

The search strategy used for MEDLINE:

#1. “Sleep apnea”[TIAB] OR “Sleep apnoea” OR “Sleep Apnea Syndromes”[MeSH] OR “Sleep apnea syndrome”[TIAB] OR “Sleep apnoea syndrome”[TIAB] OR “Sleep apnea hypopnea syndrome”[TIAB] ”Sleep apnea, Obstructive”[MeSH] OR “Obstructive sleep apnea”[TIAB] OR “Obstructive sleep apnoea” OR “Obstructive sleep apnea syndrome”[TIAB] OR “Obstructive sleep apnoea syndrome”[TIAB] OR “Obstructive sleep apnea hypopnea syndrome”[TIAB] OR “Sleep disordered breathing”[TIAB] OR “Sleep related respiratory disorder”[TIAB] OR “Sleep respiratory disorder”[TIAB].
#2. “Orthodontic appliances”[MeSH] OR “Orthodontic appliance”[TIAB] OR “Orthodontic device”[TIAB] OR “Orthodontic splint”[TIAB] OR “Oral appliance”[TIAB] OR “Oral device”[TIAB] OR “Oral splint”[TIAB] OR “Mandibular advancement appliance”[TIAB] OR “Mandibular advancement device”[TIAB] OR “Mandibular advancement splint”[TIAB] OR “Dental appliance”[TIAB] OR “Dental device”[TIAB] OR “Dental splint”[TIAB] OR “Mandibular repositioning appliance”[TIAB] OR “Mandibular repositioning device”[TIAB] OR “Mandibular repositioning splint”[TIAB] OR “Prosthetic mandibular advancement”[TIAB] OR “Mandibular Advancement/instrumentation”[MeSH].
#3. (randomized controlled trial [pt] OR controlled clinical trial [pt] OR randomized [TIAB] OR placebo [TIAB] OR clinical trials as topic [MeSH: noexp] OR randomly [TIAB] OR trial [ti]) NOT (animals [mh] NOT humans [mh]).
#4. #1 AND #2 AND #3

The search strategy used for Cochran Central Register of Controlled Trials:

#1. ((Sleep apnea) OR (Sleep apnoea) OR (Sleep apnea syndrome) OR (Sleep apnoea syndrome) OR (Sleep apnea hypopnea syndrome) OR (Obstructive sleep apnea) OR (Obstructive sleep apnoea) OR (Obstructive sleep apnea syndrome) OR (Obstructive sleep apnoea syndrome) OR (Obstructive sleep apnea hypopnea syndrome) OR (Sleep disordered breathing) OR (Sleep related respiratory disorder) OR (Sleep respiratory disorder)):ti,ab,kw
#2. ((Orthodontic appliance) OR (Orthodontic device) OR (Orthodontic splint) OR (Oral appliance) OR (Oral device) OR (Oral splint) OR (Mandibular advancement appliance) OR (Mandibular advancement device) OR (Mandibular advancement splint) OR (Dental appliance) OR (Dental device) OR (Dental splint) OR (Mandibular repositioning appliance) OR (Mandibular repositioning device) OR (Mandibular repositioning splint) OR (Prosthetic mandibular advancement)):ti,ab,kw
#3. #1 AND #2

The search strategy used for Igaku Chuo Zashi (Ichushi-Web):

#1. “Sleep apnea, obstructive”[TH] OR “Obstructive sleep apnea”[TA] OR “Sleep apnea syndrome”[TH] OR “sleep apnea syndromes”[TA] OR “Sleep apnea”[TA] OR “Sleep hypopnea”[TA] OR “sleep disordered breathing”[TA] OR “sleep related respiratory disorder”[TA] OR “Sleep respiratory disorder”[TA]
#2. “Oral appliance”[TA] OR “Oral splint”[TA] OR “Occlusal splints”[TH] OR “Sleep splint”[TA] OR “Splint”[TH] OR “Mandibular advancement device”[TA] OR “Mandibular advancement”[TA]
#3. (RD= Meta-Analysis, randomized controlled trial, controlled clinical trial, clinical guideline)
#4. #1 AND #2 AND #3

### 2.3. Study Selection Procedure

The selection of papers was made in two stages. First, two authors individually selected the articles from titles and abstracts. If in doubt, another author checked for confirmation. In the second stage, full-text papers were reviewed by two authors, and if in doubt, three authors examined them.

### 2.4. Data Extraction

Data were extracted independently by three authors. The following important information was collected: author name, year of publication, duration of the study arms, age of participants, study design, number of patients and dropouts, mean age, severity of AHI, and mean body mass index (BMI). The primary outcomes were mortality rates and cardiovascular events. Surrogate outcome #1 consisted of treatment effect, including AHI (respiratory disturbance index: RDI, respiratory event index: REI), lowest SpO_2_, arousal index, sleep efficiency, sleep stage (NREM stage 3), subjective daytime sleepiness (the Epworth Sleepiness Scale: ESS), and snoring index (SI). Surrogate outcomes #2–4 were adherence (the duration of OA usage at night and the number of days the device was used in the preceding week), sleep-related quality of life (SF-36 physical component and SF-36 mental component), and hypertension (systolic blood pressure and diastolic blood pressure), respectively. Surrogate outcome #5 consisted of side effects, including temporomandibular disorders (arthralgia or myalgia), tooth pain, occlusal changes (overbite or overjet), changes in occlusal contact, and changes in bite force. Finally, surrogate outcome #6 was patient preference. If the standard error of the mean (SEM) was reported for outcomes in the examined studies, the standard deviation (SD) was then calculated from the number of subjects in the study and the reported SEM.

### 2.5. Quality Assessment of Included Studies

The Grading of Recommendations, Assessment, Development, and Evaluation (GRADE) Approach was used to evaluate the overall quality of the evidence utilizing Review Manager 5 (Nordic Cochrane Centre, Cochrane Collaboration, 2014 Copenhagen, Denmark). Quality assessment of the study and any discrepancies were resolved by discussion among the seven authors.

## 3. Results

### 3.1. Identification and Description of Included Studies

**Figure 1:**
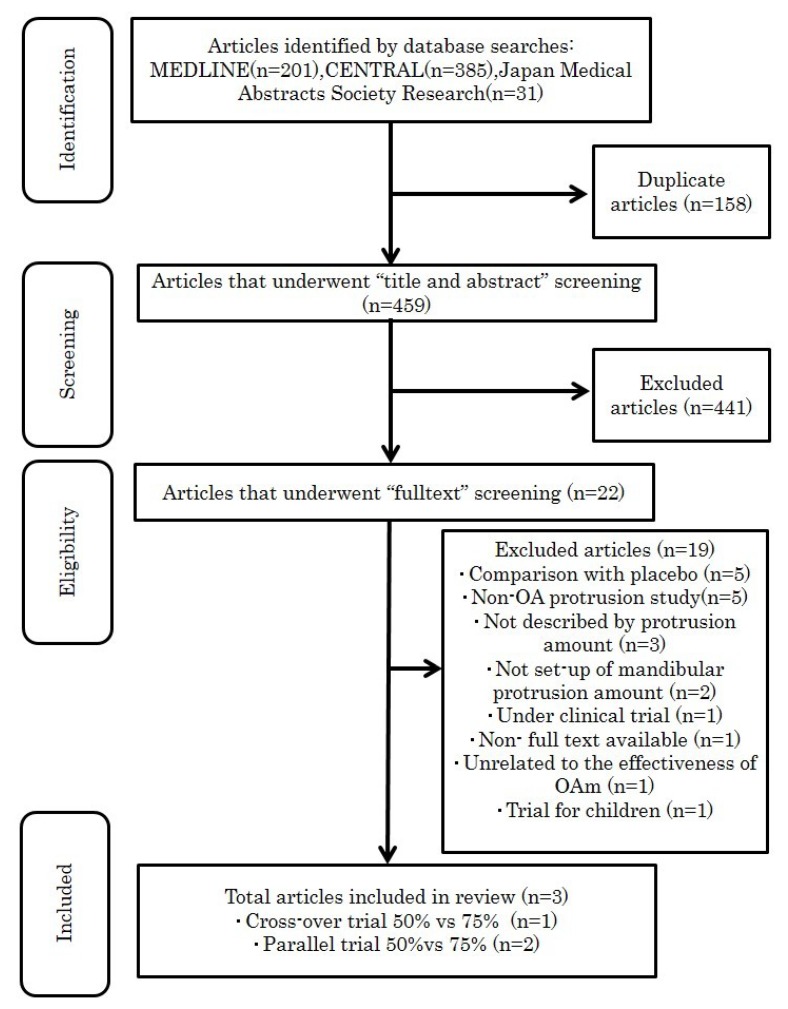
Flow diagram of the literature search.

The search identified 617 articles from the database (201 from MEDLINE, 385 from CENTRAL, and 31 from Japan Medical Abstracts Society Research) (Figure 1). After removing 158 duplicates, we conducted a title and abstract search of each study and excluded 441 articles. Twenty-two studies were retrieved for full-text assessment. Nineteen articles were excluded, as in those, comparisons were made with placebo (n = 5), OA protrusion study was absent (n = 5), protrusion amount was not disclosed (n = 3), titrating amount was not set up (n = 2), the study was under clinical trial (n = 1), no full text was available (n = 1), contradicting outcomes were observed (n = 1), and the subjects of the study were children (n = 1). The remaining three publications were included for detailed analysis. Table 1 presents the characteristic of the included studies [5,6,7].

The Aarab (2009) [5] study was a crossover trial, wherein the subject observation period was 1.5 months for the use of OAs against mild OSA. It compared 50% and 75% mandibular protrusion positions. Tegelberg (2003) [6] was a parallel trial for mild to moderate OSA over a 12-month observation period comparing 50% and 75% protrusion. Walker-Engstrom (2003) [7] was yet another parallel test in which subjects with severe OSA were observed for 6 months.

### 3.2. Meta-Analysis

**Figure 2:**
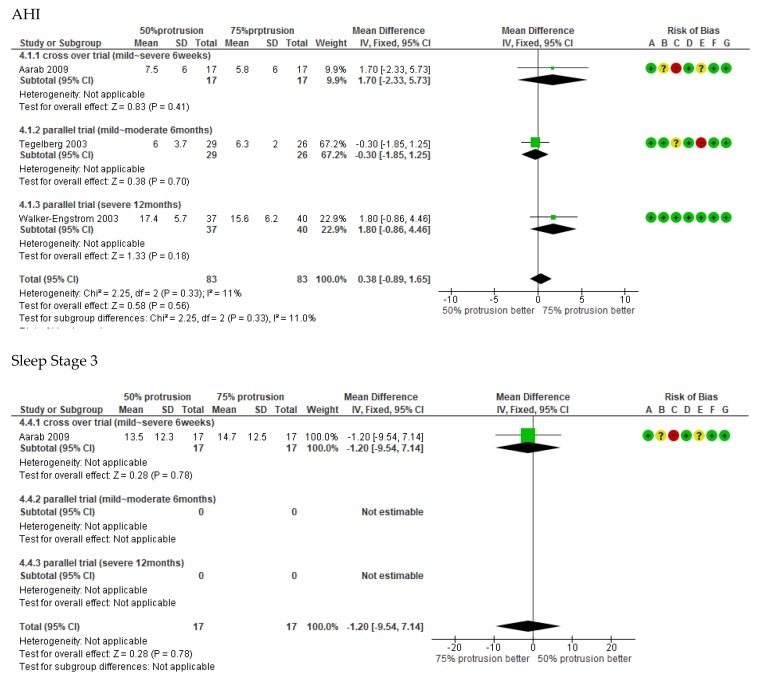

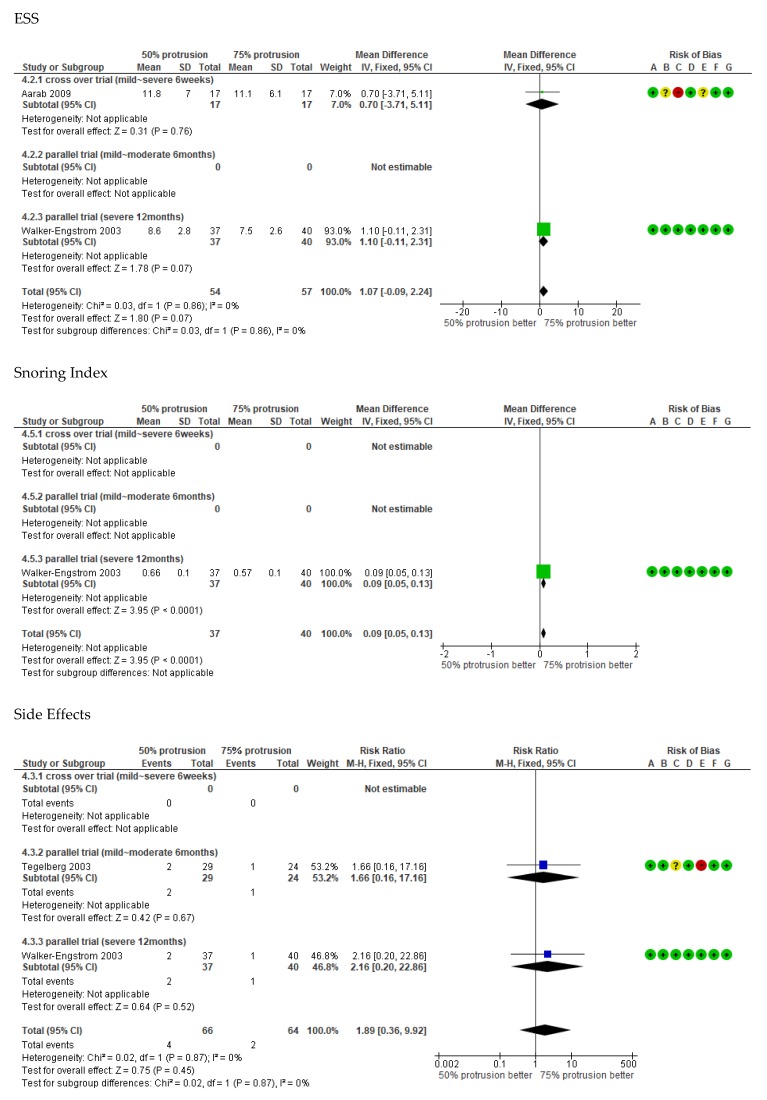
Forest plots showing the mean surrogate outcome differences between 50% protrusion and 75% protrusion in the included studies. The horizontal axis of the forest plot in Sleep Stage 3 is reversed (75%, and 50% protrusion better). Risk of bias legend: (A) Random sequence generation (selection bias), (B) Allocation concealment (selection bias), (C) Blinding of participants and personnel (performance bias), (D) Blinding of outcome assessment (detection bias), (E) Incomplete outcome data (attrition bias), (F) Selective reporting (reporting bias), and (G) Other bias.

The primary outcome of OSA treatment is the improvement of life prognosis and the prevention of cardiovascular disease. However, surrogate outcomes are often used as read outs because they require long-term research. A list of surrogate outcomes based on available reports was charted out [8,9]. As a result, there were reports on the effects of AHI, sleep stage, ESS, SI, and side effects (Figure 2). We performed subgroup analysis organized by severity of the three studies. The severity of OSA was classified by AHI (mild: 5 ≤ AHI ≺15, moderate: 15 ≤ AHI ≺ 30, severe: 30 ≤ AHI).

#### 3.2.1. Treatment Effect

Using AHI as the standard measurement, 75% protrusion is effective (0.38, 95% CI: −0.89 to 1.65; *p* = 0.58), and if classified according to severity, 75% protrusion is effective in moderate-severe OSA (1.7, 95% CI: −2.33 to 5.73; *p* = 0.41). At mild to moderate levels, 50% protrusion was effective (−0.3, 95% CI: −1.85 to 1.25; *p* = 0.70). ESS has been reviewed in two papers, and overall, 75% protrusion was more effective (1.07, 95% CI: −0.09 to 2.24; *p* = 0.07). REM sleep in stage 3 was found to be more often associated with 75% protrusion (−1.20, 95% CI: −9.54 to 7.14; *p* = 0.78), and SI improved with 75% protrusion (0.09, 95% CI: 0.05–0.13; *p* < 0.05). The horizontal axis of the forest plot in Figure 2 is (75%, and 50% protrusion better); only sleep stage 3 is reversed.

#### 3.2.2. Side Effects

Side effects were reviewed in two papers, and 75% protrusion had fewer side effects than in 50% protrusion (RR: 1.89; 95% CI: 0.36–9.92; *p* = 0.45).

### 3.3. GRADE Evidence Profile

The evidence profile was calculated using the GRADEpro software, as shown in Table 2. The quality of evidence was low due to the risk of bias and imprecision. Because the number of eligible articles was <10, a funnel plot was not used to assess publication bias in this meta-analysis.

## 4. Discussion

There are two types of OAm: the monobloc type device and the bibloc-apnea splint type. In Japan, the monobloc type is widely used and the upper and lower parts of the device are fixed with resin. Therefore, the initial position of the device is important. Many dentists set mandibular protrusion at 70% at the beginning of treatment; however, whether this position is the best is unclear. This review revealed that 75% protrusion was effective for severe OSA and 50% protrusion was effective for moderate OSA. It is thought that 50% protrusion in severe cases does not ensure a sufficiently open airway. Improvements in sleep stage 3, ESS, and SI were observed with 75% protrusion compared with 50% protrusion. OAm have better effects when a larger forward protrusion is used. An increased protrusion ensures that a wide airway is opened, thereby ameliorating drowsiness. OAm causes the lower jaw enclosure to widen, and thus, the size of the airway is increased [10]. A larger mandibular protrusion results in a larger airway opening [11]. To answer the question of why people with high OSA severity have larger forward mandibular movement, the following observations were drawn. People with high OSA severity had a BMI average value above 30 (BMI: mild OSA, 50% protrusion = 27.6 ± 3.3, mild OSA, 75% protrusion = 27.6 ± 3.0; mild to moderate OSA, 50% protrusion = 27.4 (26.4–28.4), mild to moderate OSA, 75% protrusion = 27.9 (26.6–29.3); severe OSA, 50% protrusion = 30.5 ± 1.4, severe OSA, 75% protrusion = 30.2 ± 1.2). We observed that obese patients had tongue hypertrophy, subsidence of the tongue base, and pharynx constriction [12]. Therefore, the airway can be expanded if it is further advanced. For patients with high OSA severity and high BMI, it is better for improvement of AHI to advance the mandibular more than 70%.

However, side effects such as temporomandibular disorder and discomfort may be increased. Adverse effects of OAm, such as long-term use, tooth movement, short-term temporomandibular joint, and muscle pain, have also been reported, but only a few studies seem to compare the amount of forward movement [13]. Less than 75% protrusion of the mandible is a side effect; however, as this is observed in a very small number of cases, the validity of this side effect is difficult to determine. In a clinical setting, it is common to have pain when protrusion extends to the front of the temporomandibular joint. In the present study, the comparison between the two papers was also relatively small, and the side effects due to transfer as well as the limited number of side effects should be considered in the future. Further, in some patients, even 50% protrusion in mild cases may not be sufficient; hence, it is also necessary to combine other methods to decide the most effective mandibular protrusions. Examinations such as the snoring sound test, airway evaluation using an endoscope, and cephalogram analysis can assess the presence or absence of airway dilation according to forward orientation and detect abnormalities of skeletal and soft tissues [14,15]. However, it needs to be understood that an improvement in AHI does not guarantee good OAs. A long-term study of mandibular advancement devices should examine the various potential side effects. This systematic review has some limitations. The findings are limited by the number of studies included, the relatively small number of patients studied, the period of the study and methodological weaknesses (such as blinding of participants), BMI differences, and incomplete data acquisition. Moreover, the trials were mixed parallel and crossover. It is necessary to correct the design of the trials, increase the number of patients, and have a long-term view of the possible side effects.

## 5. Conclusions

Through this review, we recommend that for patients with severe OSA, it is beneficial to begin with a mandibular protrusion of approximately 70%, and in cases of mild to moderate OSA, begin with that of approximately 50%. Further, well-designed, larger trials are required to determine the long-term benefit for patients. Specifically, investigation of adherence and side effects of long-term follow-up are needed.

## Figures and Tables

**Table 1:** Characteristics of the included studies.

Study/Year	Study Design	OSA Severity	Duration	Control Type	Number of the Beginning Patients	Number of the Patients Completed Trial	Age (Mean SD)	BMI (Mean SD)	Outcome
1, Aarab 2009	2-armed crossover; randomised	mild OSA	1.5 months	50%, 75% protrusion	50%: 20	50%: 17	50%: 51.8 (49.0–54.6)	50%: 27.6 ± 3.3	AHI, ODI, Lowest SPO_2_, Total sleep time, Stage3, Stage REM, ESS
					75%: 20	75%: 17	75%: 54.4 (52.4–56.4)	75%: 27.6 ±3.0	
2, Tegelberg 2003	2-armed Parallel; randomised	mild to moderate OSA	12 months	50%, 75% protrusion	50%: 38	50%: 29	50%: 51.8 (49.0–54.6)	50%: 27.4 (26.4–28.4)	AHI, AI, ODI
					75%: 36	75%: 26	75%: 54.4 (52.4–56.4)	75%: 27.9 (26.6–29.3)	
3, Walker-Engstrom 2003	2-armed Parallel; randomised	severe OSA	6 months	50%, 75% protrusion	50%: 42	50%: 37	50%: 54.3 (52.2–56.4)	50%: 30.5 ± 1.4	AHI, AI, ODI, SI, ESS
					75%: 42	75%: 40	75%: 50.4 (47.7–53.1)	75%: 30.2 ± 1.2	

**Table 2:** Assessment of quality using the Grading of Recommendations, Assessment, Development, and Evaluation (GRADE) system for comparison of the studies.

Certainty Assessment							No. of Patients		Effect		Certainty
No. of studies	Study Design	Risk of Bias	Inconsistency	Indirectness	Imprecision	Other Considerations	50% Protrusion	75% Protrusion	Relative	Absolute	
									(95% CI)	(95% CI)	
**AHI**											
3	randomised trials	serious ^a^	not serious	not serious	very serious ^b^	none	83	83	-	MD **0.38 higher**	VERY LOW
										(0.89 lower to 1.65 higher)	
**AHI - Cross over trial** **mild~severe** **6weeks**											
1	randomised trials	serious ^b^	not serious	not serious	very serious ^b^	none	17	17	-	MD **1.7 higher**	VERY LOW
										(2.33 lower to 5.73 higher)	
**AHI – parallel trial** **mild~moderate** **6 months**											
1	randomised trials	serious ^a,c^	not serious	not serious	very serious ^b^	none	29	26	-	MD **0.3 lower**	VERY LOW
										(1.85 lower to 1.25 higher)	
**AHI - parallel trial** **severe** **12 months**											
1	randomised trials	not serious	not serious	not serious	very serious ^b^	none	37	40	-	MD **1.8 higher**	LOW
										(0.86 lower to 4.46 higher)	
**ESS**											
2	randomised trials	not serious	not serious	not serious	very serious ^b^	none	54	57	-	MD **1.07 higher**	LOW
										(0.09 lower to 2.24 higher)	
**side effect**											
2	randomised trials	serious ^a^	not serious	not serious	very serious ^b^	none	4/66 (6.1%)	2/64 (3.1%)	**RR 1.89**	**28 more per 1000**	VERY LOW
									(0.36 to 9.92)	(from 20 fewer to 279 more)	
**Stage 3,4**											
1	randomised trials	serious ^b^	not serious	not serious	very serious ^b^	none	17	17	-	MD **1.2 lower**	VERY LOW
										(9.54 lower to 7.14 higher)	
**Snoring Index**											
1	randomised trials	not serious	not serious	not serious	very serious ^b^	none	37	40	-	MD **0.09 higher**	LOW
										(0.05 higher to 0.13 higher)	

a: the rate of dropout exceeds 20% in Tegelberg’s paper; b: the number of patients was very small.
